# A simple location-tracking app for psychological research

**DOI:** 10.3758/s13428-018-1164-y

**Published:** 2018-11-27

**Authors:** Kristoffer Geyer, David A. Ellis, Lukasz Piwek

**Affiliations:** 1 Department of Psychology, Lancaster University, Lancaster, UK; 2 School of Management, University of Bath, Bath, UK

**Keywords:** Digital traces, GPS, Location semantics, Ecological momentary assessment

## Abstract

Location data gathered from a variety of sources are particularly valuable when it comes to understanding individuals and groups. However, much of this work has relied on participants’ active engagement in regularly reporting their location. More recently, smartphones have been used to assist with this process, but although commercial smartphone applications are available, these are often expensive and are not designed with researchers in mind. To overcome these and other related issues, we have developed a freely available Android application that logs location accurately, stores the data securely, and ensures that participants can provide consent or withdraw from a study at any time. Further recommendations and R code are provided in order to assist with subsequent data analysis.

Where a person spends time can provide numerous insights into the person’s behavior, personality, and mood (Chorely, Whitaker, & Allen, 2015). For example, location measures can be predictive of depressive symptoms and levels of social anxiety (Huang et al., 2016; Palmius et al., 2017; Saeb, Lattie, Schueller, Kording, & Mohr, 2016). Other research has shown that individuals with comparable personalities often access similar locations (Noë, Whitaker, Chorley, & Pollet, 2016). While these studies remain important, critics have argued that comparatively little research has been conducted when it comes to understanding what is psychologically important about the locations people choose to occupy in real time (e.g., Rauthmann et al., 2014). Often, designs have relied on location databases harvested from social media websites (Chorley et al., 2015). However, this method presents new limitations because using social media to sample multiple locations is likely to only include the reporting of socially desirable locations (Schwartz & Halegoua, 2015). This effect may be magnified further as social media users are motivated to selectively report their location in order to maintain or boost their social status (Fitzpatrick, Birnholtz, & Gergle, 2016; Guha & Birnholtz, 2013; Schwartz & Halegoua, 2015). Similar approaches have involved self-report derived from experience sampling smartphone applications (e.g., Sandstrom, Lathia, Mascolo, & Rentfrow, 2017). However, like social media capture, the reporting of every location that an individual visits requires an extensive amount of effort. As a result, data generated from either method provide a patchy account of where a person spends their time.

Related research in medicine has also sought to understand how environmental factors influence a variety of other health outcomes (James et al., 2016). GPS data specifically, can provide highly accurate, time-stamped geographic coordinates, which link locations with environment (Müller et al., 2017). For example, trips between location points can then help quantify general levels of physical activity (Carlson et al., 2015; Jankowska, Schipperijn, & Kerr, 2015). Unfortunately, much of this research relies on the use of standalone GPS trackers, which are often expensive and may not work correctly in some buildings (Pizarro et al., 2017). In addition, standalone trackers may place a significant burden on participants who may not want to wear additional devices for extended periods of time (Schmidt, Kerr, Kestens, & Schipperijn, 2018).

Smartphones, in contrast, are readily available and used frequently by the majority of the general population (Wilcockson, Ellis, & Shaw, 2018). Advances in battery development, power management systems and location triangulation have also ensured that GPS data derived from mobile devices has become a realistic prospect (Gadziński, 2018). However, despite almost every device containing a GPS sensor, there remains a lack of suitable software that is freely available for those working within psychology and the social sciences more generally (Harari, Müller, Aung, & Rentfrow, 2017; Piwek, Ellis, & Andrews, 2016). Researchers often struggle to find appropriate alternatives from commercial application repositories—for example, via the Google Play or App stores (Apple, 2017; Google, 2017a). This is largely because these applications have not been developed with social research in mind (Table 1). Many other commercial applications often struggle to strike a suitable balance between high levels of accuracy and duration of logging, which are methodologically important for location-based research (Palmius et al., 2017). Alternative “out-of-the-box” solutions include OpenPaths (2017) and Google Timeline (Google, 2018). Although functional, OpenPaths relies on drawing data from other applications that request location updates. Therefore, data collection becomes completely under the jurisdiction of another application, and beyond a researcher’s control. Similarly, Google Timeline operates by documenting changes in location. Location is not mapped after a specific length of time, but only when a predefined distance has been covered, in order to conserve both battery and memory. Secondary data analysis derived from these systems also makes it easier for participants to omit location data from their records at any time.

**Table 1:** A comparison of the features offered by current research methods to track location from smartphones

	P	M	FTU	Features			Signal			Extra		
				AS	CR	C	GPS	Wi-fi	Ce	PI	PM	OS
PEG LOG	Android	*	*	*	*	*	*	*	*	*	*	*
AWARE (Ferreira, Kostakos, & Dey, 2015)	Android/iOS	*	*		*	*	*	*	*	*	*	*
Device Analyser (Wagner, Rice, & Beresford, 2014)	Android	*	*	*	*				*		*	
EmotionSense (Lathia, Rachuri, Mascolo, & Rentfrow, 2013)	Android		*		*	*			*		*	*
Funf (Aharony, Pan, Ip, Khayal, & Pentland, 2011)	Android		*		*	*	*	*	*	*	*	*
Lifedata (Runyan et al., 2013)	Android/iOS	*		*		*	*	*	*			
Google Timeline (Google, 2018)	Android/iOS	*	*	*	*		*	*	*	*	*	
MetricWire (2018)	Android/iOS	*		*	*	*	*	*	*	*	*	
Momento (Carter, Mankoff, & Heer, 2007)	Android		*			*			*			
MovisensXS (2018)	Android	*		*	*	*	*	*	*	*	*	
Ohmage (Ramanathan et al., 2012)	Android/iOS		*	*			*	*	*			*
OpenPaths (2017)	Android/iOS		*	*	*		*	*	*	*	*	
ResearchKit (Apple, 2016)	iOS	*	*			*	*	*	*			*
SystemSens (Falaki, Mahajan, & Estrin, 2011)	Android		*		*				*		*	*

P = platform, M = actively maintained, FTU = free to use, AS = available in app store, CR = continuous recording, C = customizable, GPS = extracts signal from global positioning system, Wi-fi = computes location from wi-fi connection, Ce = cellular location tracking, PI = assists with accurate and longitudinal point of interest mapping, PM = allows for reliable path mapping of movements, OS = open source code

To overcome these previous methodological limitations, we have developed a freely available application (*PEG LOG*) that records the location of an Android smartphone. This is an attempt to enhance the quality and quantity of data that is available to researchers when studying the significance of individual and group movements. Additionally, we wish to prompt transparency and replication by making the source code and supplementary materials freely available. Finally, the application requires minimal effort from participants, while ensuring that the associated data remain encrypted and secure throughout.

## Summary of application architecture

The application runs on Android devices and is available from the Google Play store (see the supplementary materials). It was designed to provide regular updates that circumvent the limitations associated with standalone location trackers. For example, GPS signals are typically inaccessible from inside a building, but the application can switch to rely on other available sources that report location—for example, wi-fi and network signals. However, it should be noted that both of these signals are generally less accurate than GPS alone (Android, 2018a; Canzian & Musolesi, 2015).

## Installation

**Fig. 1:**
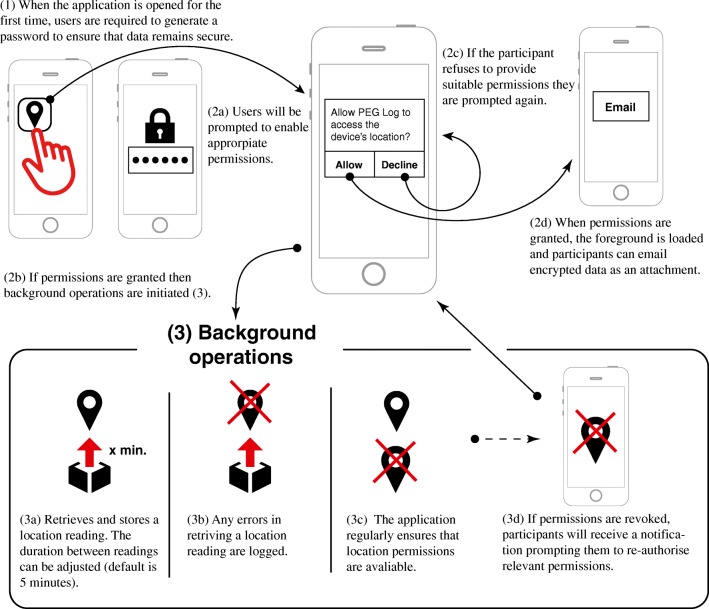
Infographic demonstrating the foreground (1, 2a–2d) and background (3a–3d) operations of the PEG LOG application. The only aspect of the application accessible by participants is the main activity page, which requests the relevant location permissions (2a) and allows participants to read the documentation, change their password, view their location data, and email files (2d).

The installation process is intended to be straightforward and requires almost no time or commitment from participants. The application must first be downloaded from the Google Play store and requires less than 30 MB of space. Once it is installed, participants simply have to open the application. This allows users to view how the application works, set a password, authorize appropriate permissions, and confirm that data collection can commence (Fig. 1). Participants will typically be asked to provide permissions related to the use of location and call data. The latter permission is required so that the application can record errors if GPS data, for example, are not available via a standard cellular connection. It is advisable that all participants send some pilot data to a researcher at the beginning of any study, to ensure that the location tracking is proceeding as expected.

## Foreground operations

### Consent and data security

Location and related behavioral data are sensitive measures, and therefore protocols must ensure that the privacy of participants’ information is protected during data collection (James et al., 2016). On first launching the application, participants are presented with a brief information screen that specifies what information is being collected and how to stop data collection. This information and the terms of service and privacy policy can be recalled at any time from within the application.^1^ The app then instructs participants to provide a six-digit password to secure their data. This password has to be communicated to the researcher in due course in order to allow any encrypted data to be accessed. It is not possible to start data collection without first defining a password. If a participant wishes to withdraw, he or she can choose not to submit the data or password. A participant can also delete files from the device by simply uninstalling the application.

To ensure that participants remain informed and aware of their active participation at all times, PEG LOG provides two visual reminders. First, the application displays a small icon in the top corner of the screen at all times. Second, a permanent text reminder will appear in the notification drawer, which explicitly states that the application is collecting location data. This has the added benefit of improving the reliability of the application as the Android operating system allocates more processing power for applications, which declare that operations are running in the background (Android, 2018b).

During the collection phase, all location data are stored in a 256-bit SQL cypher database. This ensures that even if the source code of the application were compromised, no data could be retrieved without the original password. Only PEG LOG can access this database. All exported data and associated error logs are encrypted with a 128-bit key. However, although the original password for the SQL database is fixed and cannot be changed, if the original six-digit code is forgotten, participants can modify their password via the main screen. This change will only apply to data after they have been prepared for export.

### Data storage and export

PEG LOG allows the passive recording of location data, which is stored locally on the device. This can be exported on demand. We have opted to avoid the use of a central server, in order to maximize usability; that is, researchers who want to use this app do not need to set up a cloud-based storage system, which assists with increased reliability and longevity. This also ensures that participants are in complete control of their own data throughout the entire collection process.

Data are stored in a manner that ensures that only PEG LOG can access the information. Any active email account can be used to send the file with encrypted data attached. To export data, participants can select “email” and then provide permission for the application to write to external memory, if required (Fig. 1). The data are then retrieved from the SQL cypher database and placed into an encrypted attachment. A separate encrypted error log is also provided. The application places no limit on how much data can be collected. Although it may not be possible to send larger files via email, this is unlikely to be an issue for the vast majority of studies. For example, collecting a location reading every minute for a period of two weeks generates approximately 3 MB of data.

All data are exported in PDF format, and the included R code allows these data to be unencrypted and converted to a text file quickly (see the supplementary materials). The PDF format was chosen because it can be encrypted while also allowing participants to view their own data on almost any computer or device (including smartphones). Although this may inadvertently allow participants to edit their own data, the nature and format of the location files mean that such alterations would require considerable effort. Participants who become uncomfortable with data collection or who no longer want to take part are more likely simply to uninstall the application.

## Background operations

### Recording location

The application relies on the FuseLocationProvider application (Google, 2017b). This provides access to GPS, wi-fi, and network analysis in order to retrieve latitude, longitude, accuracy levels in meters measured by a radius of confidence, and a UNIX timestamp. The application is considered high priority, which means that the most accurate reading available is provided, regardless of battery expenditure. The order of favorability of trace (in relation to accuracy) is therefore: GPS, then wi-fi, followed by network analysis (Canzian & Musolesi, 2015). A location update is requested by default every 5 min. The file returned is a lengthy table, which is stored in an SQL database (see the supplementary materials for an example of raw data). When location data cannot be collected, the application attempts to diagnose the source of the problem. For example, if the phone is restarted, this is recorded. A list of potential errors identified and their associated codes are documented in the supplementary materials. Beyond these tasks, very little processing of location data is carried out within PEG LOG itself. As a result, the application has a minimal impact on battery performance, even when the gap between location readings is comparatively small (e.g., 1-min intervals). This compares favorably with many other popular applications, which run a large a number of background processes and data-sharing mechanisms by default, which are rarely made clear to the end user (Van Kleek et al., 2017). Final decisions regarding specific data processing and analysis operations are therefore left open to researchers after data have been collected and exported from the device.

### Resilience of the application

We have identified seven potential ways that the background operations of the application could be prevented from functioning. Participants could inadvertently stop data collection by (1) turning off their phone, (2) closing the application, (3) closing all tasks running in the foreground, (4) forcing the closure of all active applications, (5) disabling location services, (6) enabling power saving modes, or (7) uninstalling the application. We address these issues in order: If the phone is turned off, upon restarting, the application will automatically resume and continue collecting data. This will be documented in internal memory and mark an interruption of data collection due to a restart event. Similarly, if the foreground section of the application is closed, the background service will continue to run. Even if all foreground applications are cleared, background services will not be interrupted. However, if a force closure of all applications occurs, then the participant will be required to open the application again in order to continue data collection. If a participant does not have location permissions enabled or if these are turned off, the application will send the participant a notification. This reminds the participant that location permissions should be enabled. Participants can click on the notification, which will point them to the relevant settings through which relevant permissions can be reenabled. In addition, the power saving modes present in some Android devices may limit the number of location points recorded by a device if it has not been used for a lengthy period of time (Android, 2018b). However, this can partly be mitigated by ensuring that participants manually whitelist the application, which increases the number of avaliable data logging windows (see the supplementary materials for more information). Finally, uninstalling the application is interpreted as a desire to withdraw from the study, and this will stop the collection of data and delete all associated files.

### Customization

Which location data source (GPS, wi-fi, etc.) is used by default, and the frequency of location updates, can be customized by following a simple modification to the original source code. This is outlined within one, nonexpert-friendly file: Constants (this file explaining the project structure is available via the associated GitHub account). Following customization, the application can then be redistributed on the Google Play store.

PEG LOG will never share data with other applications; however, the location information collected could be analyzed alongside other streams of data obtained from other applications and devices. This might include methodologies that capture time-stamped objective measures of behavior (e.g., physical activity from an accelerometer), or survey response items over longer periods of time (e.g., mood assessment from an experience sampling application) (Carlson et al., 2015; Jankowska et al., 2015; Pizarro et al., 2017).

## Storing location data

Following standard data protection guidelines, all data should be removed from email servers following transmission and stored in line with standard ethical and data protection procedures. Although data will always remain encrypted when stored on an email server, passwords should not be sent in the same email as raw data. In addition, while the application presented here remains open and freely available, location data should be treated as particularly sensitive. Researchers should keep in mind that raw and processed location data may reveal activity patterns, which participants may want to keep private (James et al., 2016). Although data can be anonymized, location coordinates are likely to reveal a persons’ place of work and home address with very little preprocessing. If these data were to be shared openly with additional anonymization, one option could involve the removal or masking of spatial data in sensitive locations (e.g., the home). Ensuring that participants understand the granularity of the data collected will help guide subsequent sharing decisions; however, more work will be required, since it is now possible to generate even larger datasets from a variety of smartphone metrics (Harari et al., 2017; Piwek et al., 2016).

## Data processing and analysis

**Fig. 2:**
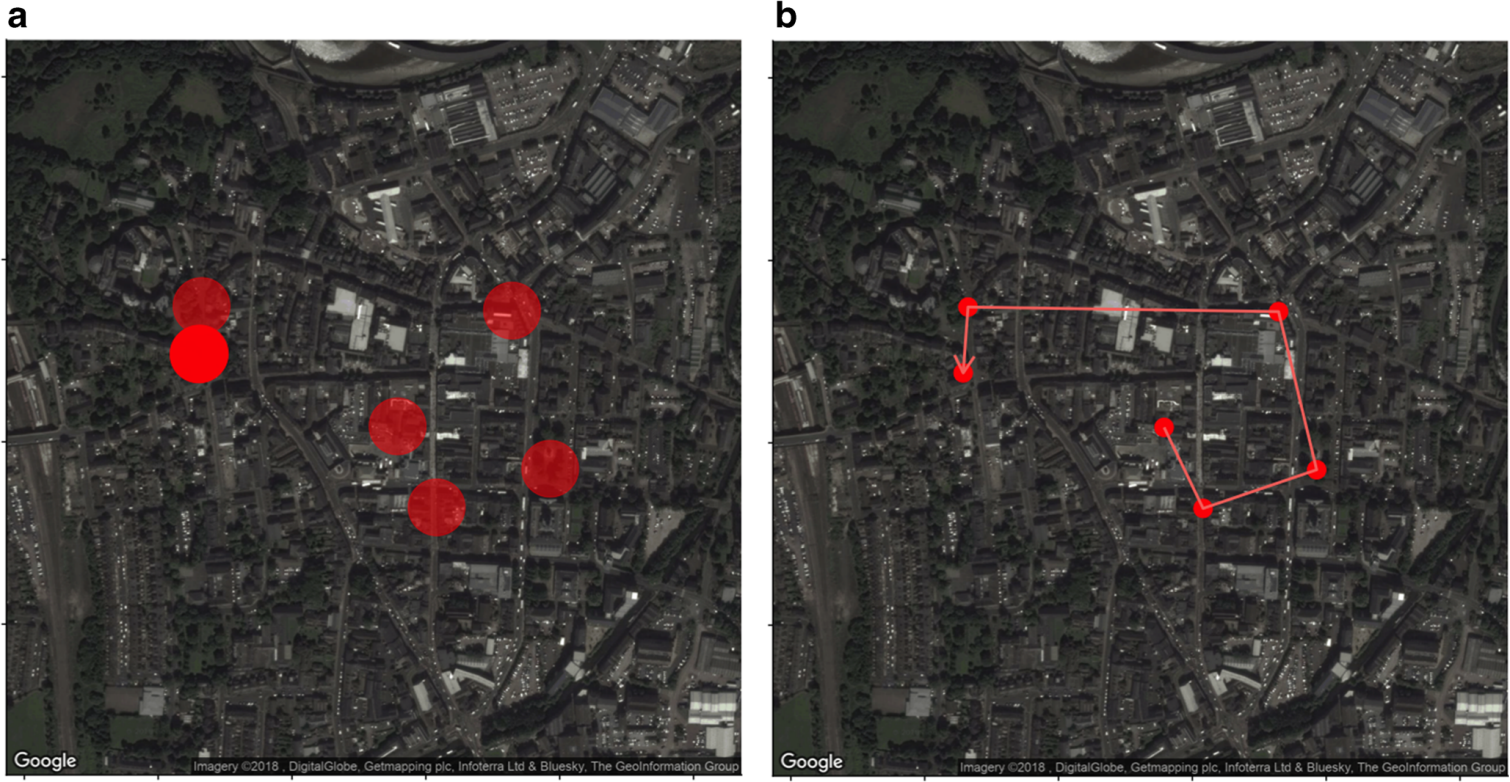
A simple visualization derived from a short period of location tracking. This includes a point map (A), which plots the individual location points (where darker points demonstrate repeated observations), and a path map (B), showing the directionality of the same participant’s movement.

A complete review concerning how location data can be analyzed is beyond the scope of this article; however, broadly speaking, there are three key ways of analyzing location data. First, location points can be placed into space-based topologies, such as cafés, university campus buildings, nightclubs, and so forth. Locations identified via this method can be further characterized on the basis of how they are clustered or relate to other geographic databases—for example, census records, crime statistics, or the foursquare database (Canzian & Musolesi, 2015; Chorley et al., 2015; Jankowska et al., 2015; Rauthmann et al., 2014). Second, movements as a form of behavior can be characterized in a number of ways (Canzian & Musolesi, 2015). This can provide information relating to distance traveled, radius of gyration, and so forth. For example, recent psychological research has shown that an analysis that includes information relating to both journey and destination is incrementally more valuable (Huang et al., 2016). Finally, a consideration of time can provide information regarding when an individual is engaged in specific activities or behaviors. For example, it is possible to separate indoor time from outdoor time (Jankowska et al., 2015). Research can, of course, combine all of these approaches; however, there remains potential for these analyses to develop further as location data become easier to collect. We have therefore included additional, supplementary R code to assist with these developments. This marked-up code will process raw location data, prepare the data for analysis, and generate some basic visualizations (Fig. 2).

## Conclusion

Previous research that has involved the collection and analysis of location data from smartphones and other digital devices has shown that this digital trace to be predictive of both future behavior and a variety of individual differences (Chorely et al., 2015). However, conclusions are often based on incomplete recordings of location from systems and devices, which are not transparent in their functionality or freely available to other researchers. Overcoming these limitations for social science remains important in order to preempt the well-documented issues with self-reported data, especially when recording location information over days, weeks, or even months (Rauthmann et al., 2014). In summary, here we have presented a freely available location-tracking application and the associated analysis code, which will allow researchers across a variety of disciplines to conduct rigorous research into individual and group movements.

### Availability of data and material

Supplementary materials, including links to the application, source code, example data, and associated R code, are available at https://github.com/kris-geyer/PEGlog.

### Author note

This work was part funded by the Centre for Research and Evidence on Security Threats [ESRC Award: ES/N009614/1]. K.G. developed and tested the application and wrote the first draft of the manuscript. D.A.E. contributed to the writing of the manuscript and the supplementary materials. L.P. also contributed to the writing of the manuscript. The authors report no conflicts of interest.

## Footnotes

1 See the supplementary materials for a link to the terms of service and privacy policy.
